# *Staphylococcus sciuri* bacteriophages double-convert for staphylokinase and phospholipase, mediate interspecies plasmid transduction, and package *mecA* gene

**DOI:** 10.1038/srep46319

**Published:** 2017-04-13

**Authors:** M. Zeman, I. Mašlaňová, A. Indráková, M. Šiborová, K. Mikulášek, K. Bendíčková, P. Plevka, V. Vrbovská, Z. Zdráhal, J. Doškař, R. Pantůček

**Affiliations:** 1 Department of Experimental Biology, Faculty of Science, Masaryk University, Kotlářská 2, 611 37 Brno, Czech Republic; 2 Central European Institute of Technology, Masaryk University, Kamenice 5, 625 00 Brno, Czech Republic; 3 Czech Collection of Microorganisms, Department of Experimental Biology, Faculty of Science, Masaryk University, Kamenice 5, 625 00 Brno, Czech Republic

## Abstract

*Staphylococcus sciuri* is a bacterial pathogen associated with infections in animals and humans, and represents a reservoir for the *mecA* gene encoding methicillin-resistance in staphylococci. No *S. sciuri* siphophages were known. Here the identification and characterization of two temperate *S. sciuri* phages from the *Siphoviridae* family designated ϕ575 and ϕ879 are presented. The phages have icosahedral heads and flexible noncontractile tails that end with a tail spike. The genomes of the phages are 42,160 and 41,448 bp long and encode 58 and 55 ORFs, respectively, arranged in functional modules. Their head-tail morphogenesis modules are similar to those of *Staphylococcus aureus* ϕ13-like serogroup F phages, suggesting their common evolutionary origin. The genome of phage ϕ575 harbours genes for staphylokinase and phospholipase that might enhance the virulence of the bacterial hosts. In addition both of the phages package a homologue of the *mecA* gene, which is a requirement for its lateral transfer. Phage ϕ879 transduces tetracycline and aminoglycoside pSTS7-like resistance plasmids from its host to other *S. sciuri* strains and to *S. aureus.* Furthermore, both of the phages efficiently adsorb to numerous staphylococcal species, indicating that they may contribute to interspecies horizontal gene transfer.

Coagulase-negative and novobiocin-resistant *Staphylococcus sciuri* is mainly considered to be a commensal, animal-associated species with a broad range of habitats including domestic and wild animals, humans and the environment^1^,^2^. The presence of the bacteria correlates with animal diseases including dog dermatitis^3^, exudative epidermitis in pigs^4^, and with a number of nosocomial diseases of humans, namely endocarditis, pelvic inflammation, and wound infections^5^,^6^,^7^,^8^. It was speculated that the *S. sciuri* species group is a potential reservoir of virulence and antimicrobial resistance genes for other staphylococci^9^. Homologues of the *mecA* gene coding for a PBP2a-like protein responsible for methicillin resistance carried on the staphylococcal cassette chromosome *mec* (SCC*mec*) are often found in bacteria from the *S. sciuri* group^10^,^11^,^12^. The protein PBP2a of *S. sciuri* has 88% amino acid sequence identity to the PBP2a of methicillin-resistant *S. aureus*^13^. The acquisition of the SCC*mec* element by methicillin-susceptible *S. aureus* was crucial for the bacterium to become a successful pathogen^14^. It is hypothesized that *S. aureus* acquired this element directly from coagulase-negative staphylococci by transduction^15^. Transduction by temperate bacteriophages is a major path for the horizontal gene transfer of virulence and resistance determinants in staphylococci^16^,^17^,^18^,^19^,^20^. In recent years, the siphovirus genomic sequences of coagulase-negative staphylococci, which are important nosocomial pathogens, have been described in detail^21^,^22^,^23^,^24^.

Here the characterization of two newly discovered *S. sciuri* bacteriophages is presented and their potential for horizontal gene transfer of the SCC elements and antibiotic resistance plasmids is analysed.

## Results

### Identification of temperate phages in *S. sciuri* species and their host range

A screening method based on the UV-induction of prophages on a 96-well tissue culture plate was applied for rapid phage detection. From the set of 15 *S. sciuri* strains, only three temperate phages from strains P575, P879 and P581 were induced and were able to propagate on the suitable host strains P612, P723 and P583, respectively (Supplementary Table S1). Phages ϕ575 and ϕ879 lysed three and five strains, respectively, out of the 36 *S. sciuri* group strains tested (Supplementary Table S2). The third phage ϕ581 induced from *S. sciuri* P581, as well as phage ϕ879, also lysed a *Staphylococcus lentus* strain, thus demonstrating an interspecies host range in oxidase-positive and novobiocin-resistant staphylococci. However, a high-titre lysate of ϕ581 could not be obtained for further analyses.

### Phage morphology and biological features

**Figure 1:**
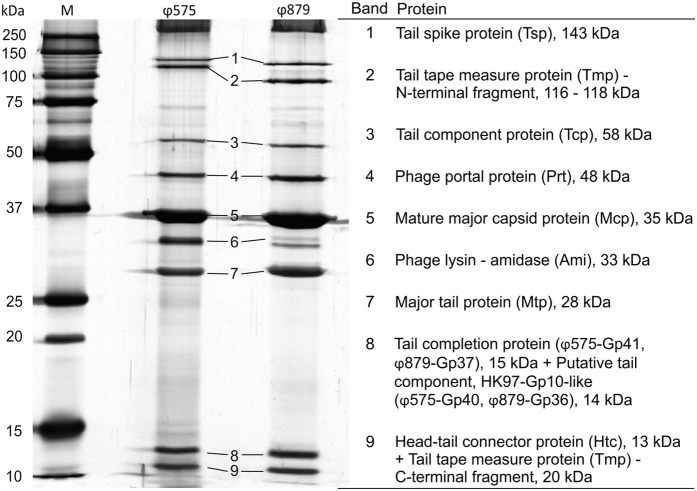
Analysis of ϕ575 and ϕ879 virion structural proteins. Left: silver-stained 1D SDS-PAGE gel with proteins extracted from purified phage particles. Selected bands were excised from the gel, digested with trypsin and subsequently analysed by mass spectrometry. Right: proteins identified by mass spectrometry with their theoretical molecular weight. M - molecular weight marker.

Both phages were able to propagate using the double-layer agar method, but not in liquid broth. They produced predominantly turbid plaques surrounded by a clear ring that indicates a high frequency of lysogenisation (Supplementary Fig. S1). Plaque diameter was variable from 0.8 to 1.4 mm. The indistinct edges of plaques that were similar for both phages may have been produced by the diffusion of lytic enzymes. The presence of endolysins in the phage lysate was confirmed by 1D SDS PAGE and subsequent mass spectrometry analysis (Fig. 1).

**Figure 2:**
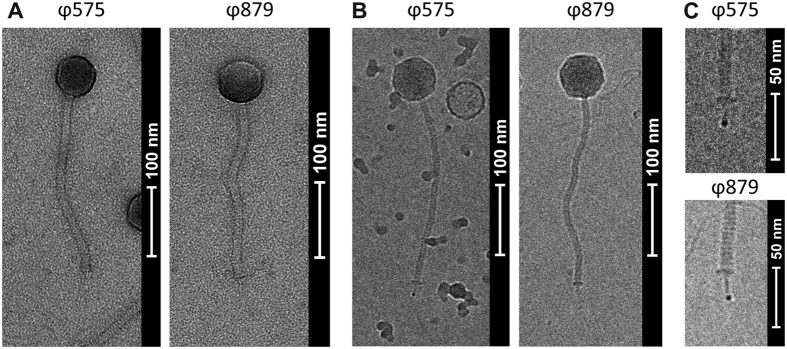
Morphology of phages ϕ575 and ϕ879. (**A**) Transmission electron microscopy images of negatively stained particles. (**B**) Cryo-electron micrographs of the phages. (**C**) Tail spike with electron-dense tip.

Morphological analysis by transmission electron microscopy (TEM) with negative staining and cryo-conditions (cryo-EM) revealed that both *S. sciuri* bacteriophages ϕ575 and ϕ879 belong to the *Siphoviridae* family. The phages consist of an icosahedral head (B1 morphology) with flexible, non-contractile tails ending with a tail spike (Fig. 2A and B). The diameters of the phage heads are 66 and 70 nm for ϕ575 and ϕ879, respectively (Supplementary Table S3). The electron density at the end of the tail spike suggests the presence of a metal ion (Fig. 2C).

**Figure 3:**
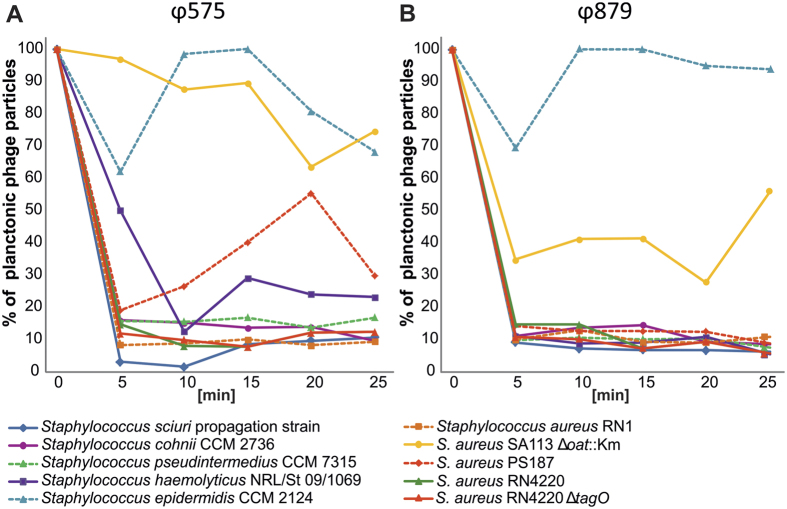
Adsorption kinetics of *Staphylococcus sciuri* bacteriophages. The adsorption rate of ϕ575 (**A**) and ϕ879 (**B**) was calculated by determining the number of unbound phage particles in the supernatant and subtracting it from the total number of input PFU. The results were expressed as a percentage of the initial phage count.

The adsorption kinetics of the phages were determined on various staphylococcal species including several *S. aureus* derivatives of laboratory isolates^25^,^26^,^27^,^28^ (Supplementary Table S1). Although both phages efficiently adsorbed onto other staphylococcal species (Fig. 3), they were unable to propagate on non-*S. sciuri* strains. The phages also adsorbed onto *S. aureus* RN4220 Δ*tagO*, which *S. aureus* siphoviruses are unable to adsorb onto due to the absence of wall teichoic acid (WTA). Phage ϕ575 adsorbed reversibly onto *S. aureus* PS 187. Both phages did not adsorb onto *S. epidermidis* CCM 2124 nor onto the *S. aureus* SA113 Δ*oat*::Km mutant, which lacks the O-acetylation of the C^6^-OH of the muramic acid of peptidoglycan.

### Genome structure

The phage genome sequences aligned with 100% identity to the prophage sequences of their lysogenic hosts. The genomes of ϕ575 and ϕ879 are arranged in functional modules and consist of 42,160 bp and 41,448 bp of dsDNA with a GC content of 31.8 and 32.1%, which is similar to the GC content of the host strains (32.4 and 32.3%). Both phage genomes have cohesive ends resulting in the formation of concatemers detected by pulsed-field gel electrophoresis (Supplementary Fig. S2). The physical ends of the phage DNA were not determined, but a significant decrease in DNA flexibility suggests that the physical ends of phage DNA are located in the region close to the 5′ end of the terminase small subunit (*terS*) gene.

Two additional prophages were identified in the strain *S. sciuri* P879. Their genomes are 43.3 and 51.0 kb in size and, like the characterized phages, have tyrosine integrase with a predicted XerC domain. Their genes encoding head proteins are arranged in a similar way to those in Mu-like phages^29^, however, attempts to induce these phages were unsuccessful.

Fifty-eight ORFs ranging from 156 bp to 4,971 bp were found in the ϕ575 genome, of which 37 have homologues in GenBank and the function was predicted for 33 of them (Supplementary Table S4). The gene density in this phage genome is 1.38 genes per kbp. The phage *att* site sequence ‘ATTCATCATAAAGTCAATACAGAACATTTGTACTTGTGCACAA’ was identified in the host strain P575 as a direct repeat between the ABC transporter *uup* gene (GenBank accession no. OFV61085.1) and a hypothetical protein gene (OFV61133.1). In the ϕ575 genome, downstream of the lytic module, the presence of virulence genes for staphylokinase (*sak*) and phospholipase (*pla2*) was detected.

The ϕ879 genome has 55 predicted ORFs ranging from 162 bp to 4,998 bp, of which 36 have homologues in GenBank and the function was predicted for 32 of them (Supplementary Table S4). Its gene density is 1.33 genes per kbp. The sequence of the phage *att* site is ‘GAATCCCTCCCAGGACGTAAATTACCAATATCCCGTTGTATCT’ and it is located between the genes for a hypothetical protein and the acetyltransferase from the GNAT family (OFV64881.1 and OFV64929.1), and overlaps with the tRNA^Arg^ gene by 17 nt (BFX02_03905). The integration module of both *S. sciuri* phages starts with the site-specific tyrosine recombinase XerC with an AP2-like DNA-binding integrase domain. The integrases themselves are dissimilar, with just 24% amino acid sequence identity.

Both phages have a σ-subunit of the RNA polymerase gene in their transcription regulation module. The presence of the sigma factor implies that both phages use their own promoters. The last protein in the DNA metabolism module is a 5-methylcytosine-specific restriction McrA-like protein that contains an HNHc endonuclease domain and is expected to provide higher resistance of their bacterial host to infection by other phages^30^. The presence of the HNH gene near the genes for the terminase subunits and portal protein is conserved in HK97-like phages^31^. The gene cluster responsible for replication exhibited similarities (66–73% nucleotide identity) to the genomes of *S. aureus* serogroup F phages such as ϕPVL or ϕN315. No predicted genes coding for functional RNAs were found in the ϕ575 and ϕ879 genomes.

**Figure 4:**
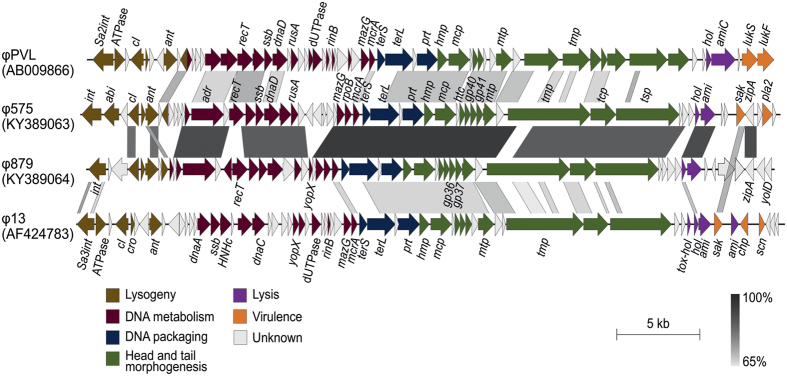
Genome comparison of *Staphylococcus sciuri* phages ϕ575 and ϕ879 and *Staphylococcus aureus* phages ϕPVL and ϕ13. The GenBank accession number for each sequence is given in brackets. Genomes were aligned using the blastn algorithm and similar regions with more than 65% identity are indicated. The positions and orientations of the coding regions are represented by arrows. Genome modules are color-coded according to the legend.

The DNA packaging module follows the DNA metabolism module, and the two phages share 97% nucleotide identity in this module. The highest similarity (65–73% and 61–74% nucleotide identity) in the packaging and head module of the ϕ575 and ϕ879 phages was found to the *S. aureus* triple-converting phage ϕ13 from serological group F^32^ from the Sa3 integrase family, and the *S. aureus* Panton-Valentine converting phage ϕPVL^33^ from the Sa2 integrase family (Fig. 4). The module for head-tail morphogenesis encodes structural virion proteins and is also highly similar in both of the described phages (90% nucleotide identity).

Structural proteins were analysed by SDS-PAGE in combination with mass spectrometry (Fig. 1). The major capsid protein (Mcp) belongs to the HK97 family. Its 120-amino acid-long N-terminal δ domain is cleaved by prohead protease and is not present in mature virions of the analysed phages. Mass spectrometry showed a 94% coverage of mature Mcp (Supplementary Fig. S3). The molecular weight of Mcp determined from SDS-PAGE gel is ~35 kDa (Fig. 1). Tail tape measure protein (Tmp), which determines the length of the tail, tends to be proteolytically cleaved^34^. The C-terminal end of Tmp contains a lysozyme-like domain involved in the hydrolysis of beta-1,4-linked polysaccharides of the bacterial cell wall and was detected in mature phages (Fig. 1). The function of the second largest protein in this module was predicted to be a tail spike protein determined on the basis of its position, length and secondary structure forming numerous beta sheets. No other tail-associated cell wall hydrolases were detected in the phage proteomes.

The module responsible for lysis of the bacterial cell is almost identical for both phages (Fig. 4). The holins of ϕ575 and ϕ879 are conserved in both phages and share over 90% amino acid identity. They exhibit more than 60% amino acid identity to *S. aureus* and *Staphylococcus pseudintermedius* prophage holins. The endolysins of ϕ575 and ϕ879 exhibit a low similarity to well-characterized staphylococcal endolysins and lack a cell-wall binding domain.

### Horizontal transfer of pSTS7-like plasmid between *S. sciuri* and *S. aureus* strains

While analysing the sequencing data from purified phages, reads that map to bacterial and plasmid DNA sequences were found. These findings implied that phages are able to package non-phage DNA. Phage ϕ879 was able to package a 5,667-bp-long pSTS7-like plasmid designated pSSC723 from propagation strain P723. This plasmid encodes tetracycline efflux protein (TetL) and aminoglycoside adenyltransferase (AadD). The ratio between the plasmid-borne *aadD* gene and the reference tail protein gene determined by qPCR was 5.7 × 10^−3^. Plasmid pSSC723 belongs to the pC194 family and exhibits almost 100% nucleotide identity to the intergene spacer from the previously described *S. epidermidis* plasmid pSTS7. Plasmid pSTS7 originated from the interplasmid recombination of *Bacillus subtilis* plasmid pNS1981 and *S. aureus* plasmid pUB110^35^.

The transducing abilities of phage ϕ879 were verified by the transfer of pSSC723 to *S. sciuri* strains P600 and P574, and *S. aureus* strain RN4220. The donor strain P723 with plasmid pSSC723 grew on selective plates with tetracycline and kanamycin, whereas the recipients did not. Rare transductants with a frequency of about 10^−11^ were obtained in two intraspecies and one interspecies transduction to *S. aureus*. Plasmid identity in the donor strain and in transductants was confirmed by sequencing, and the genetic background of transductants was confirmed by macrorestriction analysis with *Sma*I by PFGE (Supplementary Fig. S4).

### Packaging of *S. sciuri* SCC*mec* element

Both sequencing reads and quantification using real-time PCR proved the ability of phage ϕ879 to package *mecA* and plasmid genes from its bacterial host into virions. The packaging frequency of the *mecA* part of SCC*mec* was 3 × 10^−5^ as determined by qPCR analysis. Cassette chromosome recombinase (*ccr*) genes A, B and C were detected in phage ϕ879 particles using endpoint PCR. However, the successful transduction of SCC*mec* into recipient *S. aureus* strains was not observed.

**Figure 5:**
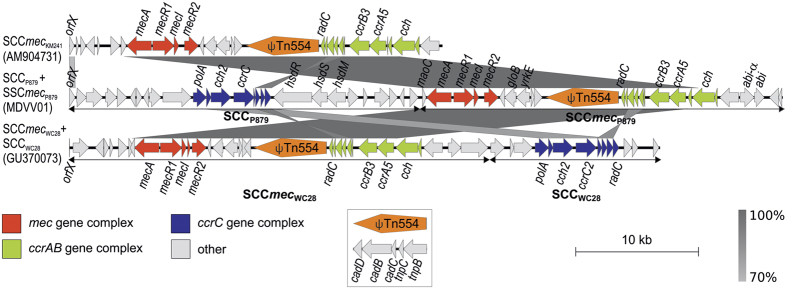
Visualization of SCC*mec*_P879_ element nucleotide sequence alignment to related SCCs. Sequences of SCC elements from *Staphylococcus pseudintermedius* KM241, *Staphylococcus sciuri* P879 and *Staphylococcus cohnii* WC28 were aligned using the blastn algorithm and similar regions with more than 70% identity are indicated. The GenBank accession number for each sequence is given in brackets. The positions and orientations of coding regions are represented by arrows.

A novel SCC*mec*_P879_ element whose parts packaged to phage ϕ879 virions was identified in methicillin-resistant prophage host *S. sciuri* strain P879. The SCC*mec*_P879_ element is 29,546 bp long, harbouring a *mec* A-class complex and *ccrB3* and *ccrA5* recombinases, and was integrated into the consensus insertion sequence site for SCC elements (GAAGCTTATCATAAGTAA). The major part (21 kb) of the SCC*mec*_P879_ shared 98% nucleotide identity with the SCC*mec* element of *S. pseudintermedius* strain KM241^36^ and the SCC*mec* element of *Staphylococcus cohnii* strain WC28^37^. However their J1 and J3 regions were different (Fig. 5). Ccr recombinases in SCC*mec*_P879_ exhibited 90–96% amino acid identity to recombinase complexes identified previously in *S. sciuri*^38^ or *S. cohnii*^37^. A cluster of genes including the *cch* gene for MCM-like helicase, which is conserved in a number of other SCC elements, was identified downstream of the recombinase genes *ccrB3* and *ccrA5*. In addition, a non-*mec* SCC element designated SCC_P879_, which is 21,844 bp long, was identified adjacent to SCC*mec*_P879_. SCC_P879_ carries the *ccrC* recombinase gene, genes for a restriction-modification type I system and heavy metal resistance genes (Fig. 5).

In methicillin-susceptible *S. sciuri* P575, a non-SCC homologue of the *mecA* gene (WP_070369365.1) was identified, coding for a PBP2a-like protein with 95% amino acid identity to PBP2a from *S. aureus*. This gene is located between a hypothetical protein gene (WP_058611716.1) and an amino acid permease gene (WP_070369378.1) and was also quantified in phage particles. The relative ratio between *mecA* and the reference tail protein gene copy numbers in phage particles was 8.2 × 10^−4^, which is higher than for phage ϕ879.

## Discussion

Many prophages reside in the bacterial genomes and contribute to bacterial evolutionary processes via the horizontal exchange of genetic information^40^. Some prophages confer novel biological properties to their host strains, enabling them to adapt to new environments or obtain virulence determinants by lysogenic conversion, thereby driving bacterial adaptation and evolution. Typically, 1–4 prophages are found in each *S. aureus* genome^41^. Similarly, putative prophages often reside in the genomes of some coagulase-negative staphylococci^42^ and *Macrococcus caseolyticus* related to *S. sciuri*^43^, but only a few *S. epidermidis, Staphylococcus hominis* and *Staphylococcus capitis* viable phages have been genetically characterized in detail^21^,^22^,^23^. Recently, *S. sciuri* myoviruses isolated from urban sewage were described^44^, however no *S. sciuri* siphoviruses have been morphologically and genetically characterized to date.

The genomes of *S. sciuri* phages ϕ575 and ϕ879 have modular structures and sizes that are similar to those of other staphylococcal siphoviruses^21^,^32^,^45^,^46^. The phage genomes share 76% overall nucleotide identity, as calculated by a global alignment algorithm. Since they are related to *S. aureus* serogroup F phages belonging to integrase type Sa3, it is suggested that both bacteriophages belong to the *Biseptimavirus* genus^47^. Additional similarities with putative prophages across the genus *Staphylococcus* were identified in a Blast search of the microbial genome database. ϕ575 and ϕ879 exhibit 66–72% and 73–80% nucleotide identity, respectively, in their head-tail modules with prophages present in the genomes of *S. pseudintermedius* strains 063228 and NA45^48^ and *Staphylococcus schleiferi* strains 5909–02 and 2317–03^49^.

Even though the phages are similar, each integrates to different *att* sites in the host chromosome. This is because of differences in their integration modules. The integrase from ϕ879 has 51% amino acid identity to the Sa3 integrase from related *S. aureus* phage ϕ13, mainly in the C-terminal catalytic domain. The phage integrase from ϕ575 has 34% amino acid identity to the integrases from the *Lysinibacillus* and *Bacillus* spp. phages^50^.

Virulence factor-converting phages have not been detected in coagulase-negative staphylococci yet. Staphylokinase is a plasminogen activator protein secreted by most human lysogenic *S. aureus* strains. A secondary function of staphylokinase is its ability to neutralize host antimicrobial peptides whose binding may control its plasminogen activation properties^52^. The staphylokinase of ϕ575 exhibits a 52% amino acid sequence identity to that of *S. aureus* phages encoded by the immune evasion cluster associated with Sa3 integrase family phages^53^,^54^.

Phospholipase homologues can influence the innate and adaptive immune response and damage the membranes of host cells^55^. Phages carrying a gene for phospholipase were identified in streptococci^56^. Both virulence factors Sak and Pla2 contain an approximately 30-amino acid-long signal peptide that is required for protein secretion. The lysogenic hosts carrying phospholipase or staphylokinase are more likely to become virulent. There are two ORFs with opposite orientation between the *sak* and *pla2* genes in the ϕ575 phage genome. The first, *zipA*-like gene was located on the complementary strand and the second gene has unknown function but was predicted to contain a transmembrane domain. The organisation of virulence factors in this region is similar to that of the immune evasion cluster, which harbours the *sea, sak, scn*, and *chp* genes^54^. Therefore the cluster of genes following the lysis module in ϕ575 could include other potential virulence factors.

Bacteriophages with a tail spike structure bind to proteinaceous receptors^57^. Prototypical phages with a tail spike are lactococcal bacteriophage p2^58^ and *B. subtilis* phage SPP1^59^. The high electron density observed at the end of the tail spike of ϕ575 and ϕ879 might be due to the presence of metal ions as was previously shown for the tail of bacteriophage p2, where iron, calcium and chloride ions were identified in the spike’s trimeric metal-binding structure^60^,^61^.

It is generally assumed that *pac* phages, which include the *S. aureus* phages of serogroup B, are responsible for generalized transduction^62^. In contrast, here it was shown that *cos* phages ϕ575 and ϕ879 package host genes in their virions. This finding corroborates previous observations of the transmission of pathogenicity islands via *cos* phages^63^,^64^.

Two types of mobile genetic elements, SCC*mec* and an pSTS7-like plasmid, were detected by qPCR in the phage ϕ879 particles. The SCC*mec*_P879_ core genes detected in ϕ879 virions are highly similar to SCC elements from other staphylococcal species such as *S. pseudintermedius* and *S. cohnii*. This indicates a possible horizontal interspecies transfer of this element. The MCM-like helicase identified in SCC*mec*_P879_ possibly participates in the extrachromosomal replication of this element^65^. This may increase the probability of SCC*mec* packaging into the phage virions. The relative quantity of packaged plasmid DNA in phage ϕ879 particles was higher than the relative quantity of the *mecA* gene from the SCC*mec* region. This implies that the pSSC723 plasmid is packaged as a multimer, as was suggested for other small plasmids^66^. In addition, plasmid interspecies and intraspecies transfer was proved at low frequencies, although the effective infection of *S. sciuri* phage ϕ879 has not been observed in the recipient strains. two recent studies, it was confirmed that phage adsorption on the recipient cell wall is sufficient for successful transduction, and effective infection is not necessary^18^,^20^.

The effective adsorption of analysed phages on the cells of non-*S. sciuri* species (Fig. 3) supports the speculated interspecies horizontal transfer of mobile elements or their parts by phages. A reversible adsorption of both phages was observed in *S. aureus* PS 187 that has a distinct structure of WTA^28^, suggesting the possibility of a two-stage adsorption. This type of adsorption has been described for the *B. subtilis* SPP1 phage as a result of a missing YueB membrane receptor on mutant host cells, where the glucosylated poly (glycerol-phosphate) of cell WTA proved to be a major target for SPP1 reversible binding^67^. A homologue of the YueB receptor described in *B. subtilis* was not found in staphylococci, however membrane proteins homologous to the phage infection proteins of the PIP family (e.g. YhgE) may act as possible receptors. The successful adsorption of ϕ575 and ϕ879 to *S. aureus* knockout mutant RN4220 Δ*tagO* and also the inability of both phages to adsorb onto *S. aureus* SA113 Δ*oat* suggests that teichoic acids are not their only receptors, as with *S. aureus* phages^68^, but that O-acetyl groups at the 6-position of muramic acid play a role in the process of adsorption.

## Conclusions

The identification of bacterial virulence factors encoded by *S. sciuri* phages, their ability to package and transmit mobile elements and to adsorb onto the cells of other staphylococcal species show that *S. sciuri* siphoviruses may contribute to the horizontal gene transfer within the *Staphylococcus* genus. Additionally, phages ϕ575 and ϕ879 exhibit evolutionary relationships to phages of the coagulase-positive species *S. aureus* and *S. pseudintermedius*, indicating a possible common gene pool of the phage genomes of these pathogens.

## Material and Methods

### Bacterial strains and Bacteriophages

Fifteen *S. sciuri* strains were selected for the screening of temperate phages and an additional 21 strains from the *S. sciuri* species group were used for the host-range determination. All *S. sciuri* strains were obtained from the National Reference Laboratory for Staphylococci (NRL/St), National Institute of Public Health (Prague, Czech Republic) and characterized previously^2^. The presence of the phages and their host range were tested on the set of *S. sciuri* strains by soft agar spot assay. The phage host strains, propagation strains, and strains used in the characterization of phage biological properties are listed in Supplementary Tables S1 and S2. The adsorption efficiencies of the phages were determined as described previously^18^. The antibiotic resistance pattern of selected *S. sciuri* strains was tested by the disc diffusion method on Mueller-Hinton agar (Oxoid) with discs (Oxoid) generally used for Gram-positive cocci (Supplementary Table S5).

### High-throughput detection of UV-inducible prophages

A modified protocol designed for prophage induction on 96-well plates was used in this study^69^. Twenty μl of the overnight cultures were dispensed in a 96-well culture plate with 180 μl of CM1 nutrient broth (Oxoid, United Kingdom). The plates were incubated for 2 hours at 37 °C with shaking (120 rpm). After centrifugation at 1,100 g for 15 min, the pellet was resuspended in 200 μl of physiological solution and the plates were UV-irradiated for 25 s or 35 s using a 15 W UV-lamp (340 nm) at a distance of 60 cm. Forty μl of the irradiated suspension was transferred to a new plate together with 120 μl of physiological solution and 40 μl of 10× concentrated prophage broth per well, consisting of 10 g Trypton L42 (Oxoid), 2 g Yeast extract powder L21 (Oxoid), 2 g NaCl and 13 g Nutrient broth CM1 (Oxoid) dissolved in 100 ml of distilled water. The plates were protected from daylight and incubated at 37 °C for 2 hours. The plates were then centrifuged at 2,000 g for 10 min and the supernatants were passed through a 0.45 μm filter (TPP Techno Plastic Products, Switzerland) into a sterile microtube. If the screening resulted in lysis of the indicator strains, the presence of viable phages was proved by large-scale UV or mitomycin C induction.

### Phage propagation and purification

Phages obtained from a single plaque were propagated using a double-layer agar technique with 1.5% 2YT bottom agar and 0.7% top Agar No. 1 L11 (Oxoid) with the addition of CaCl_2_ to a concentration of 2 mM. After overnight incubation, the top layer was disrupted and the phage was washed down with broth, centrifuged twice for 30 min at 3,100 g and filtered through a 0.45-μm filter. Phage particles were purified in a CsCl density gradient^70^.

### Electron and Cryo-electron microscopy

Negative-stained samples were prepared by double staining in 2% uranyl acetate. Cryo samples were prepared by vitrification of the bacteriophage solution (at a concentration of 10^10^ PFU ml^−1^) on Quantifoil grids by plunging into liquid ethane using an FEI Vitrobot Mark IV. All samples were observed with an FEI Tecnai F20 electron microscope operated at 200 kV at a magnification of 29,000×.

### Plasmid transduction

Intraspecies and interspecies transduction experiments were performed as described previously^18^. Transductants were selected on plates with kanamycin and tetracycline (both at concentration 8 μg ml^−1^), where the recipient strains *S. sciuri* P600, P574 and *S. aureus* RN4220 were unable to grow.

### DNA extraction from the phage particles

DNase I (Sigma-Aldrich, Germany) and RNase A (Sigma-Aldrich) treatment was performed to remove any exogenous host genomic DNA and RNA from purified phage particles as described previously^70^. The DNA was isolated with a Phage DNA Isolation Kit (Norgen Biotek Corporation, Canada) according to the manufacturer’s recommendations. DNA from the viral particles for sequencing was isolated by phenol-chloroform extraction^62^. The concentration and purity of the phage DNA was determined using a NanoDrop spectrophotometer (Thermo Fisher Scientific, USA).

### Pulsed-field gel electrophoresis

One μg of phage DNA was loaded onto 1.5% agarose gel and separated by PFGE (Cheff Mapper, Bio-Rad, USA) to detect concatemerized cohesive ends. A constant voltage of 5 V cm^−1^ and switch times of 2-20 s with linear ramping were applied. Lambda DNA concatemers and a 5 kb DNA ladder (Bio-Rad) were used as molecular weight markers.

### Genome sequencing and bioinformatic analysis

Phage genome and bacterial whole-genome shotgun (WGS) sequencing was performed using an Ion Torrent™ Personal Genome Machine (Ion PGM™). The purified genomic DNA was used for preparing a 400-bp sequencing library with an Ion Plus Fragment Library Kit (Thermo Fisher Scientific). The sample was loaded on a 316v2 chip and sequenced using an Ion PGM Hi-Q sequencing kit (Thermo Fisher Scientific). Quality trimming and error correction of the reads were performed with the Ion Torrent Suite Software (version 5.0.2). The assembly computation was performed using the implemented Assembler SPAdes (v.3.1.0) with default parameters for Ion Torrent data.

Sequences were manipulated and inspected in the cross-platform bioinformatics software Ugene v.1.23.1^71^. The primal analysis of sequences was a combination of open reading frames (ORFs) prediction using GeneMark.hmm^72^ and automatic annotation by RAST^73^. Gene content was further examined via a BLASTp search on protein sequence databases^74^, CD-Search^75^ and InterPro v.59^76^. tRNAscan-SE^77^, RNAmmer v.1.2^78^, and hmmsearch v.3.0^79^ were used to analyse functional RNAs. Protein secondary structure was predicted with JPred4^80^. Multiple sequence alignments were visualized using EasyFig v.2.1^81^.

### Nucleotide sequence accession numbers

The complete genomes of the *S. sciuri* phages ϕ575 and ϕ879 were deposited in GenBank under the accession numbers KY389063 and KY389064, respectively. The data from the WGS of bacterial host strains P575 and P879 were recorded in the GenBank WGS project under the accession numbers MDVU01 and MDVV01, respectively. The sequence of the pSSC723 plasmid was deposited in GenBank under the accession number KY389065.

### SDS-PAGE and Mass Spectrometry

Vertical one-dimensional electrophoresis (1-DE), using Bio-Rad equipment, was performed in a Protean II xi Cell (discontinuous 12% T SDS-PAGE). Precision Plus Protein Standard (Bio-Rad) was applied as the molecular weight marker. Proteins were stained with a ProteoSilver Plus kit (Sigma Aldrich). Protein bands were excised, destained and subjected to tryptic digestion (40 °C, 2 h) without the reduction and alkylation of cysteine residues. Digested peptides were extracted from gels using 50% acetonitrile solution with 2.5% formic acid and concentrated in a SpeedVac concentrator (Thermo Fisher Scientific). LC-MS/MS analyses of the peptide mixture were done using an RSLCnano system (Thermo Fisher Scientific) on-line connected to an Impact II Ultra-High Resolution Qq-Time-Of-Flight mass spectrometer (Bruker, Bremen, Germany). Prior to LC separation, tryptic digests were on-line concentrated in a trap column. The peptides were separated using an Acclaim Pepmap100 C18 column (3 μm particles, 75 μm × 500 mm; Thermo Fisher Scientific, 300 nl min^−1^) and a 0.1% FA/acetonitrile gradient. MS data were acquired in a data-dependent strategy with a 3 s cycle time. Mass range was set to 150–2,200 m/z and precursors were selected from 300 to 2,000 m/z. Mascot (version 2.4.1) MS/MS ion searches were done against a local database containing translated phage insert sequences. Mass tolerance for precursors and MS/MS fragments were 15 ppm and 0.05 Da, respectively. The oxidation of methionine, deamidation (N, Q) and propionamide (C) were set as variable modifications for all searches.

### The relative quantification analysis of bacterial genes in phage particles

The target genes for qPCR were first detected by conventional end-point PCR and the amplicons verified by sequencing. A LightCycler 490 Real-Time PCR Instrument (Roche Diagnostics, USA) was used for relative quantification analysis. Reactions were carried out in triplicates in MicroAmp^®^ optical 96-well reaction plates sealed with optical adhesive covers (Roche Diagnostics). Each reaction mixture (10 μl) contained 7.5 μl FastStart^®^ TaqMan Probe Master (Roche Diagnostics), 900 nM of each primer, 250 nM of hydrolysis probe (Supplementary Table S6), and 50 ng of template DNA. An initial denaturation of DNA at 95 °C for 10 min was followed by 40 cycles of amplification (95 °C for 15 s and 60 °C for 45 s).

The relative quantification analysis was performed as a dual-colour experiment. The FAM probes were used to target *mecA* and aminoglycoside acetyltransferase genes, the Cy5 probe was used for reference phage gene coding for the head-tail connector protein. The results were generated by the Light-Cycler software using the maximum of the second derivative, and a target gene was paired with the reference by a one-to-one pairing analysis. The reaction efficiency was calculated for each analysed gene, and the results were normalized according to the efficiencies from the relative quantification. The calculated ratio between the number of target gene copies and reference gene copies was estimated.

## Additional Information

**How to cite this article**: Zeman, M. *et al. Staphylococcus sciuri* bacteriophages double-convert for staphylokinase and phospholipase, mediate interspecies plasmid transduction, and package *mecA* gene. *Sci. Rep.* **7**, 46319; doi: 10.1038/srep46319 (2017).

**Publisher's note:** Springer Nature remains neutral with regard to jurisdictional claims in published maps and institutional affiliations.

## Supplementary Material

Supplementary Material
