# Supplementary material for: Staphylococcus sciuri bacteriophages double-convert for staphylokinase and phospholipase, mediate interspecies plasmid transduction, and package mecA gene
Source: Sci Rep. 2017 Apr 13;7:46319. doi: 10.1038/srep46319 (PMC5390265; doi:10.1038/srep46319)
Supplement: Supplementary Material [file srep46319-s1.pdf]

Staphylococcus sciuri bacteriophages double-convert for staphylokinase and phospholipase, mediate interspecies plasmid transduction, and package *mecA* gene

M. Zeman[1], I. Mašlaňová[1], A. Indráková[1], M. Šiborová[2], K. Mikulášek[2], K. Bendíčková[3], P. Plevka[2], V. Vrbovská[1,3], Z. Zdráhal[2], J. Doškař[1] and R. Pantůček*[1]

[1]Department of Experimental Biology, Faculty of Science, Masaryk University, Kotlářská 2, 611 37 Brno, Czech Republic. [2]Central European Institute of Technology, Masaryk University, Kamenice 5, 625 00 Brno, Czech Republic. [3]Czech Collection of Microorganisms, Department of Experimental Biology, Faculty of Science, Masaryk University, Kamenice 5, 625 00 Brno, Czech Republic.

Supplementary Table S1. Characteristics of strains used in this study.

Strain	Strain description	Reference
S. sciuri P575	φ575 host strain, human origin (urine)	Švec *et al.*, 2016
S. sciuri P879	φ879 host strain, human origin (wound swab)	Švec *et al.*, 2016
S. sciuri P581	φ581 host strain, human origin (blood culture)	Švec *et al.*, 2016
S. sciuri P612	propagation strain for φ575, human origin (urine)	Švec *et al.*, 2016
S. sciuri P723	propagation strain for φ879, animal origin (piglet), pSSC723 plasmid	Švec *et al.*, 2016
S. lentus P583	susceptible strain to φ581, human origin (blood culture)	this study
S. sciuri P574	plasmidless transduction recipient strain, human origin (urine)	Švec *et al.*, 2016
S. sciuri P600	plasmidless transduction recipient strain, human origin (urine)	Švec *et al.*, 2016
S. cohnii CCM 2736	type strain of *S. cohnii*, used in adsorption kinetics studies	CCM
S. pseudintermedius CCM 7315	type strain of *S. pseudintermedius,* used in adsorption kinetics studies	CCM
S. haemolyticus NRL/St 09/1069	clinical isolate used in adsorption kinetics studies, human origin (blood culture)	this study
S. epidermidis CCM 2123	type strain of *S. epidermidis,* used in adsorption kinetics studies	CCM
S. aureus RN1	strain NCTC 8325, used in adsorption kinetics studies	
S. aureus RN4220	prophageless derivate of 8325-4, *agr*+ background, 11-bp deletion in *rsb*U, restriction-defective, used in adsorption kinetics studies and as transduction recipient	Kreiswirth *et al.*, 1983
S. aureus RN4220 Δ*tagO*	gene knockout mutant deficient in the peptidoglycan-anchored wall teichoic acid synthesis, used in adsorption kinetics studies	Xia *et al.*, 2011
S. aureus SA113 Δ*oat*::Km	derivate of strain 8325, gene knockout mutant (Δ*oat*::kan) deficient in the peptidoglycan O-acetylation, *agr*- background, 11-bp deletion in *rsbU*	Bera *et al.*, 2005
S. aureus PS187	propagation strain for phage 187, poly-glycerol-phosphate (GroP) WTA glycosylated with N-acetyl-D-galactosamine (GalNAc), used in adsorption kinetics studies	Winstel *et al.*, 2014

Legend: CCM, Czech Collection of Microorganisms (http://www.sci.muni.cz/ccm/) has provided type cultures.

Suplementary Table S2. Sensitivity of bacterial strains from the *S. sciuri* complex to bacteriophages φ575 and φ879. Phage suspensions containing to 10^4 PFU/ml were used for typing. Unless stated otherwise, the strains are of human origin.

<table>
<tr><th>Strain [1]</th><th>Year of isolation</th><th>Source</th><th>φ575</th><th>φ879</th></tr>
<tr><td>P548</td><td>1997</td><td>Pustule, dog</td><td>not sensitive</td><td>rare various plaques</td></tr>
<tr><td>P612</td><td>1999</td><td>Urine</td><td>various plaques</td><td>rare turbid plaques</td></tr>
<tr><td>P723</td><td>2003</td><td>Piglet</td><td>rare various plaques</td><td>clear plaques</td></tr>
<tr><td>P583</td><td>2000</td><td>Blood culture</td><td>not sensitive</td><td>rare turbid plaques</td></tr>
<tr><td>P537</td><td>1995</td><td>Urine</td><td>clear plaques</td><td>not sensitive</td></tr>
<tr><td>P536</td><td>1995</td><td>Burn</td><td rowspan="31">not sensitive</td><td rowspan="31">not sensitive</td></tr>
<tr><td>P539</td><td>1997</td><td>Furuncle</td></tr>
<tr><td>P540</td><td>1997</td><td>Drain</td></tr>
<tr><td>P543</td><td>1997</td><td>Eye</td></tr>
<tr><td>P545</td><td>1998</td><td>Urine</td></tr>
<tr><td>P549</td><td>1998</td><td>Urine</td></tr>
<tr><td>P553</td><td>1998</td><td>Urine</td></tr>
<tr><td>P554</td><td>1998</td><td>Urine</td></tr>
<tr><td>P556</td><td>1998</td><td>Urine</td></tr>
<tr><td>P563</td><td>1999</td><td>Mastitis, cow</td></tr>
<tr><td>P568</td><td>1999</td><td>Urine</td></tr>
<tr><td>P569</td><td>1999</td><td>Urine</td></tr>
<tr><td>P572</td><td>1999</td><td>Nose, Norway rat</td></tr>
<tr><td>P574</td><td>1999</td><td>Urine</td></tr>
<tr><td>P575</td><td>2000</td><td>Urine</td></tr>
<tr><td>P578</td><td>2000</td><td>Urine</td></tr>
<tr><td>P581</td><td>2000</td><td>Blood culture</td></tr>
<tr><td>P589</td><td>2001</td><td>Urine</td></tr>
<tr><td>P597</td><td>2001</td><td>Catheter</td></tr>
<tr><td>P600</td><td>2002</td><td>Urine</td></tr>
<tr><td>P601</td><td>2002</td><td>Food</td></tr>
<tr><td>P602</td><td>2002</td><td>Urine</td></tr>
<tr><td>P605</td><td>2002</td><td>Wound</td></tr>
<tr><td>P607</td><td>2002</td><td>Drain</td></tr>
<tr><td>P609</td><td>2002</td><td>Decubitus</td></tr>
<tr><td>P611</td><td>2003</td><td>Haemoculture</td></tr>
<tr><td>P879</td><td>2003</td><td>Wound</td></tr>
<tr><td>P880</td><td>2003</td><td>Vagina</td></tr>
<tr><td>CCM 3473 [2]</td><td>1973</td><td>Skin, squirrel</td></tr>
<tr><td>CCM 4657</td><td>1992</td><td>Nose, Norway rat</td></tr>
<tr><td>CCM 4835</td><td>1991</td><td>Food</td></tr>
</table>

[1] Strains belong to *S. sciuri* species, except P583 and P597 that belong to *S. lentus* species

[2] Type strain of *S. sciuri* species

Supplementary Table S3. Dimensions of the phages on the basis of electron-microscopy analysis.
The results are an average from 10 independent measurements.

Microscopy technique	Bacteriophage	Head diameter [nm]	Tail length [nm]	Tail width [nm]
Transmission electron microscopy	φ575	60.4 ± 0.72	255.4 ± 2.39	9.6 ± 0.17
	φ879	65.7 ± 1.57	247.5 ± 1.69	9.7 ± 0.64
Cryo-electron microscopy	φ575	65.8 ± 1.54	261.4 ± 2.59	10.2 ± 0.56
	φ879	69.7 ± 1.41	261.4 ± 5.13	9.3 ± 0.47

Supplementary Table S4. Genome annotation of *Staphylococcus sciuri* phages φ575 and φ879.

**Genome annotation of phage φ575** (GenBank accession no. KY389063)						
Gene no.	Start	End	Length [nt]	Strand	Predicted function	Gene identifier
gp1	75	1256	1182	-	Phage integrase	*int*
gp2	1396	2325	930	-	Abortive infection bacteriophage resistance protein	*abi*
gp3	2435	2839	405	-	Phage hypothetical protein	
gp4	2900	3514	615	-	Phage cI-like repressor with peptidase and LexA domain	*cI*
gp5	3686	3913	228	+	Phage HTH-type transcriptional regulator	
gp6	3937	4713	777	+	Phage antirepressor protein	*ant*
gp7	4726	4920	195	+	Phage hypothetical protein	
gp8	4907	5287	381	-	DUF2513 domain-containing phage protein	
gp9	5335	5640	306	+	Phage hypothetical protein	
gp10	5647	5904	258	+	Phage hypothetical protein	
gp11	5879	6079	201	+	Phage hypothetical protein	
gp12	6127	6306	180	+	Phage hypothetical protein, predicted transmembrane	
gp13	6307	6612	306	+	DUF1108 domain-containing phage protein	
gp14	6699	8651	1953	+	ATPase involved in DNA repair, phage associated	*adr*
gp15	8648	8983	336	+	Phage hypothetical protein	
gp16	8980	9879	900	+	Recombinational DNA repair protein RecT(prophage associated)	*recT*
gp17	9975	10577	603	+	Phosphoribosyl phosphodiesterase - like protein in prophage	
gp18	10578	11060	483	+	Single-stranded DNA-binding protein	*ssb*
gp19	11093	12022	930	+	Phage replication initiation protein, DnaD	*dnaD*
gp20	12006	12713	708	+	DUF881 domain-containing phage protein	
gp21	12707	13132	426	+	Phage Holliday junction resolvase, RusA-like	*rusA*
gp22	13132	13530	399	+	Phage hypothetical protein	
gp23	13606	14013	408	-	Phage hypothetical protein	
gp24	14023	14547	525	-	Phage hypothetical protein, predicted non-cytoplasmatic	
gp25	14629	14799	171	+	Phage hypothetical protein, predicted non-cytoplasmatic	
gp26	14871	15287	417	+	Phage hypothetical protein	
gp27	15268	15477	210	+	Phage hypothetical protein	
gp28	15464	15922	459	+	Putative NTP pyrophosphohydrolase MazG family	*mazG*
gp29	15956	16357	402	+	Putative phage RNA polymerase sigma 70 factor, region 3/4	*rpoD*
gp30	16448	16846	399	+	Putative phage 5-methylcytosine-specific restriction endonuclease McrA-like protein	*mcrA*
gp31	17018	17500	483	+	Phage terminase, small subunit	*terS*
gp32	17484	19211	1728	+	Phage terminase, large subunit	*terL*

**Genome annotation of phage φ879** (GenBank accession no. KY389064)						
Gene no.	Start	End	Length [nt]	Strand	Predicted function	Gene identifier
gp1	143	1171	1029	-	Phage integrase	*int*
gp2	1343	1531	189	+	Phage hypothetical protein	
gp3	1505	2476	972	-	Phage hypothetical protein	
gp4	2535	3155	621	-	Phage cI-like repressor with peptidase and LexA domain	*cI*
gp5	3300	3539	240	+	Phage HTH-type transcriptional regulator	
gp6	3573	4355	783	+	Phage antirepressor protein	*ant*
gp7	4369	4533	165	+	Phage hypothetical protein	
gp8	4545	4937	393	+	Phage antirepressor protein, BRO family	
gp9	5038	5265	228	+	DUF771 domain-containing phage protein	
gp10	5262	5441	180	+	Phage hypothetical protein, predicted transmembrane	
gp11	5442	5747	306	+	DUF1108 domain-containing phage protein	
gp12	5835	7787	1953	+	ATPase involved in DNA repair, phage associated	*adr*
gp13	7784	8119	336	+	Phage hypothetical protein	
gp14	8282	8752	471	-	DUF2321 domain-containing phage protein	
gp15	8816	9724	909	+	Recombinational DNA repair protein RecT (prophage associated)	*recT*
gp16	9820	10422	603	+	Phosphoribosyl phosphodiesterase - like protein in prophage	
gp17	10423	10905	483	+	Single-stranded DNA-binding protein	*ssb*
gp18	10938	11867	930	+	Phage replication initiation protein, DnaD	*dnaD*
gp19	11851	12558	708	+	Phage hypothetical protein	
gp20	12555	12977	423	+	Phage Holliday junction resolvase, RusA-like	*rusA*
gp21	12977	13375	399	+	Phage hypothetical protein	
gp22	13436	13651	216	+	YopX-like protein	*yopX*
gp23	13645	13854	210	+	Phage hypothetical protein	
gp24	13841	14299	459	+	Putative NTP pyrophosphohydrolase MazG family	*mazG*
gp25	14333	14734	402	+	Putative phage RNA polymerase sigma 70 factor, region 3/4	*rpoD*
gp26	14825	15223	399	+	Putative phage 5-methylcytosine-specific restriction endonuclease McrA-like protein	*mcrA*
gp27	15393	15875	483	+	Phage terminase, small subunit	*terS*
gp28	15859	17586	1728	+	Phage terminase, large subunit	*terL*
gp29	17598	17798	201	+	Phage hypothetical protein, predicted transmembrane	
gp30	17804	19069	1266	+	Phage portal protein, HK97-like	*prt*
gp31	19116	19658	543	+	Phage head maturation protease, HK97 family	*hmp*
gp32	19768	20994	1227	+	Phage major capsid protein, HK97-like	*mcp*

gp33	19223	19423	201	+	Phage hypothetical protein, predicted transmembrane	
gp34	19429	20694	1266	+	Phage portal protein, HK97-like	*prt*
gp35	20741	21283	543	+	Phage head maturation protease, HK97 family	*hmp*
gp36	21392	22618	1227	+	Phage major capsid protein, HK97-like	*mcp*
gp37	22661	22855	195	+	Phage hypothetical protein	
gp38	22867	23199	333	+	Phage DNA packaging, Head-Tail Connector Protein HK97 Gp6-like	*htc*
gp39	23183	23521	339	+	Putative phage head-tail adaptor	
gp40	23521	23898	378	+	Structural, putative tail-component HK97-Gp10-like	
gp41	23895	24275	381	+	Structural protein, putative tail component	
gp42	24288	25004	717	+	Phage major tail protein	*mtp*
gp43	25080	25529	450	+	Phage hypothetical protein	
gp44	25779	30749	4971	+	Structural, tail tape measure protein	*tmp*
gp45	30764	32284	1521	+	Structural, tail component protein	*tcp*
gp46	32300	36100	3801	+	Structural phage protein, putative tail spike with endopeptidase domain	*tsp*
gp47	36097	36252	156	+	Phage hypothetical protein	
gp48	36290	36631	342	+	Phage hypothetical protein	
gp49	36681	36980	300	+	DUF2951 domain-containing phage protein	
gp50	37069	37317	249	+	Phage holin	*hol*
gp51	37403	38272	870	+	Phage lysin, N-acetylmuramoyl-L-alanine amidase	*ami*
gp52	38629	38787	159	+	Antitoxin-like protein	
gp53	38812	39042	231	+	Phage hypothetical protein	
gp54	39551	40057	507	+	Phage-encoded staphylokinase	*sak*
gp55	40086	40586	501	-	ZipA protein N-terminal domain	*zipA*
gp56	40826	41098	273	+	Phage hypothetical protein, predicted transmembrane	
gp57	41117	41722	606	+	Phospholipase A2	*pla2*
gp58	41726	41932	207	+	Phage hypothetical protein, predicted membrane anchored	

gp33	21037	21231	195	+	Phage hypothetical protein	
gp34	21243	21575	333	+	Phage DNA packaging, Head-Tail Connector Protein HK97 Gp6-like	*htc*
gp35	21565	21897	333	+	Bacteriophage SPP1, head-tail adaptor-like protein	
gp36	21897	22274	378	+	Structural, putative tail-component HK97-Gp10-like protein	
gp37	22271	22651	381	+	Structural protein, putative tail component	
gp38	22664	23380	717	+	Phage major tail protein	*mtp*
gp39	23456	23905	450	+	Phage hypothetical protein	
gp40	24156	29153	4998	+	Structural, tail tape-measure protein	*tmp*
gp41	29168	30688	1521	+	Structural, tail component protein	*tcp*
gp42	30704	34507	3804	+	Structural phage protein, putative tail spike with endopeptidase domain	*tsp*
gp43	34507	34668	162	+	Phage hypothetical protein	
gp44	34709	34996	288	+	Phage hypothetical protein	
gp45	35037	35498	462	+	Phage hypothetical protein, predicted transmembrane	
gp46	35498	35884	387	+	Phage hypothetical protein, predicted membrane anchored	
gp47	35897	36157	261	+	Phage holin	*hol*
gp48	36233	37102	870	+	Phage lysin, N-acetylmuramoyl-L-alanine amidase	*ami*
gp49	37282	37458	177	+	Phage hypothetical protein	
gp50	37717	37917	201	-	Phage hypothetical protein	
gp51	38098	39099	1002	+	DUF4868 domain-containing phage protein	
gp52	39111	39668	558	+	Phage hypothetical protein, predicted transmembrane	
gp53	39727	40227	501	-	Cell division protein ZipA	*zipA*
gp54	40382	40882	501	-	Phage hypothetical protein, predicted membrane anchored	
gp55	40893	41303	411	-	YolD-like protein	*yolD*

Supplementary Table S5. Antimicrobial susceptibility profile of *S. sciuri* phage hosts and propagation strains. The testing was performed by disc diffusion method on Mueller Hinton agar. EUCAST clinical breakpoints V. 6.0 (http://www.eucast.org/clinical_breakpoints/) were used to interpret susceptibility testing.

Antibiotic	Disc (Oxoid)	Prophage host strain		Phage propagation strain	
		P575	P879	P612	P723
Cefoxitin	FOX30	S	R	S	S
Oxacillin	OX1	S	R	S	S
Penicillin G	P1	R	R	R	R
Ciprofloxacin	CIP5	S	S	S	S
Clindamycin	DA2	R	R	R	R
Erythromycin	E15	S	S	S	S
Trimethoprim+Sulfamethoxazole	SXT25	S	S	S	S
Fusidic acid	FD10	R	R	R	R
Gentamicin	CN10	R	R	R	R
Rifampicin	RD5	S	S	S	S
Tigecycline	TGC15	S	S	S	S
Mupirocin	MUP200	R	R	S	R
Tetracycline	TE30	S	S	S	R
Chloramphenicol	C30	S	S	S	S
Linezolid	LZD10	S	S	S	S
Vancomycin	VA5	S	S	S	S
Kanamycin	K30	S	R	S	R
Novobiocin	NV5	R	R	R	R

S, susceptible; R, resistant

Supplementary Table S6. Primers and probes used for analyses of abilities of phages to package and transduce bacterial DNA.

Target gene	Sequence of primers and probes
Aminoglycoside adenyltransferase (*aadD*)	FW: 5' CATCTGTGCCAGTTCGTA 3'<br>RV: 5' AAATTCTCTAGCGATTCCAG 3'<br>Probe: FAM- 5' CTCAGAGTCGGAAAGTTGACC 3'
Penicillin-binding protein (*mecA*)	FW: 5' AGYGTCATYATTCCAGG 3'<br>RV: 5' CYACATTRTTTCGGTCT 3'<br>Probe: FAM- 5' CGTTCTGATTTCAATGGTTCAA 3'
Head-tail connector protein (*htc*)	FW: 5' TAAAAGCTTATTATGAGTGGG 3'<br>RV: 5' TAATAGTACGCTGTTAGTGGA 3'<br>Probe: Cy5- 5' ATTCATCAGTTACTGCACTCG 3'
Cassette chromosome recombinases A, B *(ccrAB)*	FW: 5' GAAATACCTCTATCCGCGTA 3'<br>RV: 5' TAAACTTATTACCCGCAAGCC 3'
Cassette chromosome recombinase (*ccrC)*	FW: 5' TTATCAAAATTGGTCCGCTA 3'<br>RV: 5' GTAGTTCATCATTACGGGTT 3'

A φ575

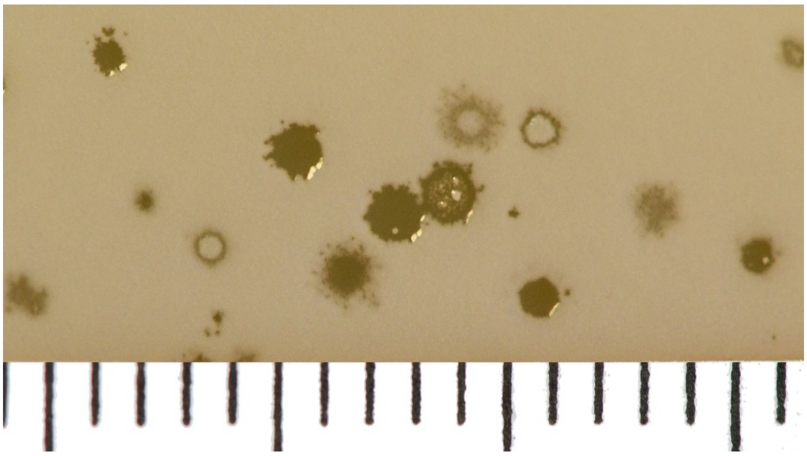

B φ879

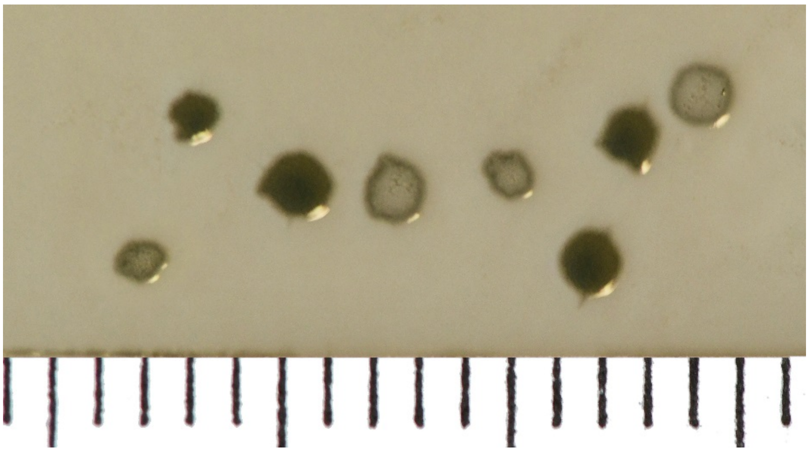

Supplementary Figure S1. Plaque morphology of *Staphylococcus sciuri* phages φ575 (A) and φ879 (B). The phage plaque assay was performed using the double-layer agar method on 2YT Agar. The ruler scale is in millimetres.

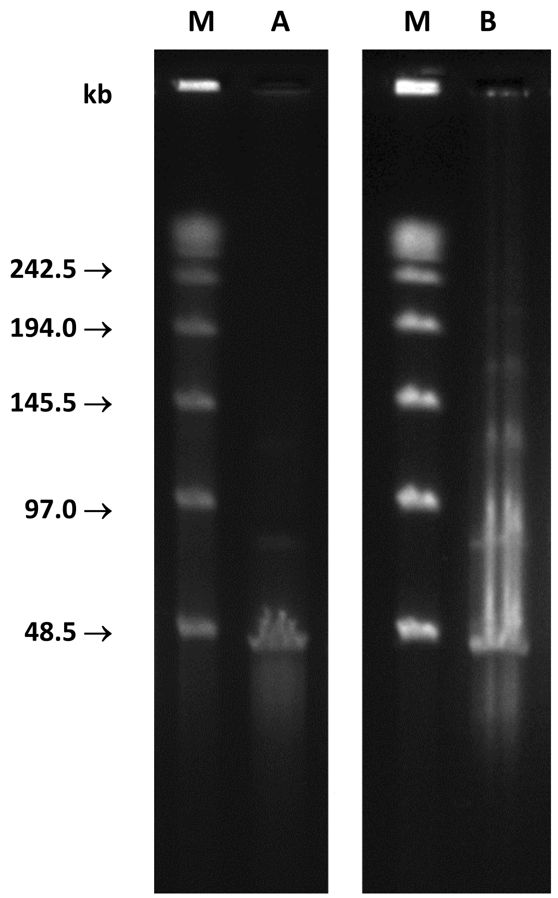

Supplementary Figure S2. Pulsed-field gel electrophoresis of bacteriophage DNA showing concatemer formation of phage genomic DNA as a result of interactions between cohesive ends. Lane M, Lambda DNA concatemers (Bio-Rad); Lane A, DNA of phage φ575; Lane B, DNA of phage φ879.

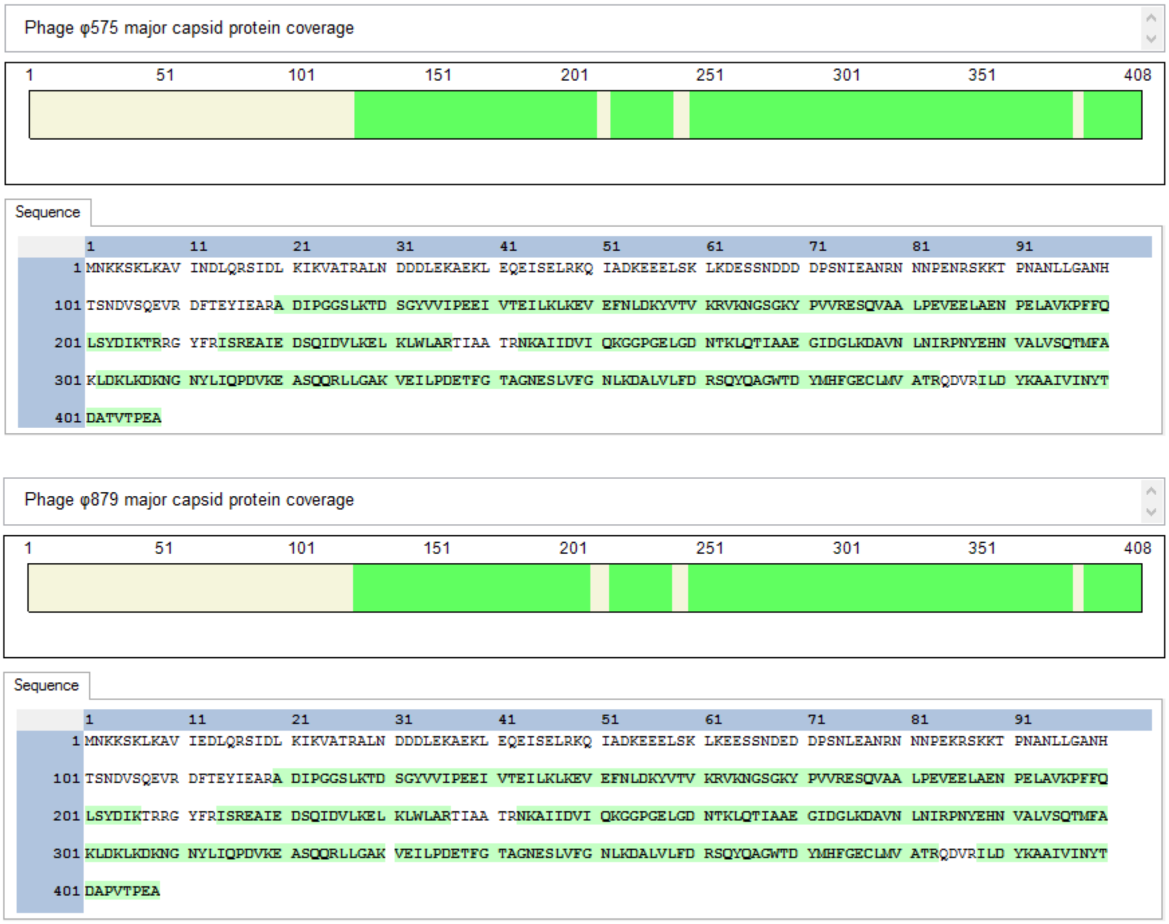

Supplementary Figure S3. Sequence coverage of major capsid proteins of phages φ575 and φ879. Coverage of major capsid protein sequence is highlighted in green for both phages. Data evaluation was done using Proteome Discoverer v. 1.4 (Thermo Fisher Scientific). The sequence coverage is based purely on significant peptide hits ($p < 0.01$). Other filter criteria included: lowest peptide length (6 amino acid) and use only the best identified peptides for given peptide spectrum matches (rank 1).

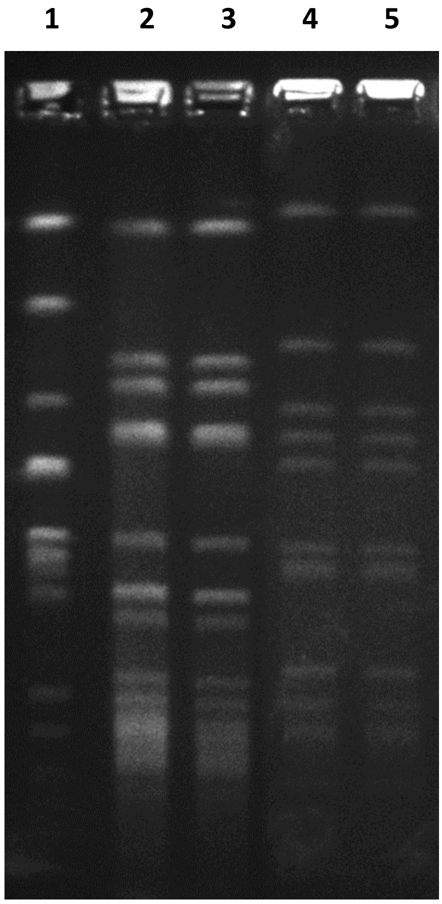

Supplementary Figure S4. Pulsed-field gel electrophoresis analysis of transductants. The genetic background of interspecies and intraspecies transductants proved by *Sma*I macrorestriction analysis was in agreement with that of the recipient strains. Lane 1, donor strain *S. sciuri* P723 harbouring plasmid pSSC723; Lane 2, recipient strain *S. aureus* RN4220; Lane 3, transductant *S. aureus* RN4220::pSSC723; Lane 4, recipient strain *S. sciuri* P600; Lane 5, transductant *S. sciuri* P600::pSSC723.
